# How does young adults’ dietary and health-related quality of life vary by food security and household income?

**DOI:** 10.3389/fnut.2024.1505771

**Published:** 2024-12-19

**Authors:** Eun-kyung Kim, Yong-Seok Kwon, Sena Kim, Jin-Young Lee, Young Hee Park

**Affiliations:** National Institute of Agricultural Sciences, Rural Development Administration, Wanju, Republic of Korea

**Keywords:** young adults, Korea National Health and Nutrition Examination Survey (KNHANES), food security, household income, Euro Quality of Life-Five Dimensions (EQ-5D)

## Abstract

**Objectives:**

The objective of this study was to compare the dietary and health-related quality of life of young adults according to their household income and food security status.

**Methods:**

To conduct this study, 10,224 young adults aged 19–34 years who participated in the 2008–2018 Korea National Health and Nutrition Examination Survey (KNHANES) were selected. Subjects were categorized into four groups based on household income and food security: ‘food secure and high income,’ ‘food insecure and high income,’ ‘food secure and low income,’ and ‘food insecure and low income’. General characteristics, daily diet, and dietary quality were compared among the four groups.

**Results:**

The proportion of participants consuming a daily diet below the estimated average requirements for protein, vitamins A, B_1_, and B_2_, niacin, vitamin C, calcium, phosphorus, and iron increased in the ‘food insecure and low income’ group. Among the most commonly consumed foods, instant noodles and Sprite ranked relatively high in the ‘food insecure and low income’ group, while apples and beef ranked relatively high in the ‘food secure and high income’ group. The food insecure and low income group exhibited significantly increased rates of mobility problems [OR = 1.55(95% CI = 1.05–2.29)] and anxiety/depression [OR = 1.33(95% CI = 1.07–1.64)] in comparison with the food secure and high income group.

**Conclusion:**

Food insecurity was positively associated with poor diet quality and was associated with health-related quality of life, mobility, and anxiety/depression, especially among young adults.

## 1 Introduction

The term ‘household food insecurity’ is used to describe a situation in which a household lacks both the availability of and access to sufficient food, due to economic difficulty or other insufficient resources (1, 2). Food insecurity remains a significant concern not only in low- and middle-income countries but also in high-income countries (3–5). There is a growing body of evidence indicating a relationship between food insecurity and a range of socio-economic characteristics (6), including asthma (7), health-related quality of life (HRQoL) (8), body composition (9), underweight and poverty among older adults (10), obesity (11), sleep complaints (12), and cardiovascular disease (13).

The prevalence of food insecurity is dependent on a number of factors, including age, ethnicity, and region. The prevalence of food insecurity among adults aged 19–64 years in Korea was 8.2% (11.3% for individuals aged 1 or older) (14). In the United States, it is estimated that 13.5% (equivalent to 18.0 million households) experienced food insecurity at least some time throughout the entire year in 2023 (15). Furthermore, 5.1% (6.8 million households) exhibited very low food security. In the Mexican population-based survey, the prevalence of mild, moderate, and severe food insecurity was 41, 20, and 12%, respectively (16).

Food insecurity is identified as a significant risk factor for mental health outcomes, including depression and anxiety (17). A report by the WHO indicates that depression was the primary cause of global disability in 2015, accounting for 7.5% of cases, while anxiety disorders ranked sixth with a prevalence of 3.4%. The global prevalence of depression was estimated at 322 million cases (4.4%), with the highest prevalence observed in the South-East Asian region, accounting for 27% (85.67 million) of the global total. The global prevalence of anxiety disorders was estimated at 264 million (3.6%), with the South-East Asia region accounting for the highest number, at 23% (60.05 million) (18). The Euro Quality of Life-Five Dimensions (EQ5D) is an index devised to measure health-related quality of life, including anxiety/depression, mobility, self-care, usual activities, and pain/discomfort (19). It has been reported to have a relationship with diet quality (20–23), food insecurity (8, 24), and chronic diseases (25). Furthermore, it is presented as a predictor of mortality in older adults (26).

In the Korean population, several studies have been conducted on the prevalence of food insecurity (27) and the associations between food insecurity and dietary intake (14, 28–31), childhood obesity (32), asthma (7), and risk factors in older adults (33). Nevertheless, there has been a paucity of research conducted on young adults in comparison with other age groups, as well as a dearth of studies examining the relationship between food insecurity and health-related quality of life in this demographic. Therefore, the objective of this study was to evaluate the dietary quality and health-related quality of life in a vulnerable young adult population using data from the Korea National Health and Nutrition Examination Survey (KNHANES).

## 2 Methods

### 2.1 Data source and study population

This study was based on data from the Korea National Health and Nutrition Survey (KNHANES), a cross-sectional, nationally representative survey conducted by the Korea Disease Control and Prevention Agency^1^. The study was approved by the Institutional Review Board of the Korea Centers for Disease Control and Prevention (2008-04EXP-01-C, 2009-01CON-03-2C, 2010-02CON-21-C, 2011-02CON-06-C, 2012-01EXP-01–2C, 2013-07CON-03–4C, 2013-12EXP-03-5C, and 2018-01-03-P-A). KNHANES is a legally mandated survey conducted in accordance with the provisions of the Korean National Health Promotion Act. Its objective is to assess the prevalence of health-related behaviors, the incidence of chronic diseases, and the nutritional state of foodstuffs among the Korean population. In accordance with Article 2–1 of the Bioethics and Safety Act and Article 2–2-1 of the Enforcement Regulations of the same Act, KNHANES is a research project conducted for the benefit of the public. Consequently, it was conducted in 2015–2017 with IRB review exemption. The survey targeted 11,250 adults aged 19–34 years who participated in the health survey and the dietary intake survey of KNHANES IV-2 to KNHANES VII-3. The exclusion criteria were as follows: individuals lacking food security data (*n* = 30), individuals lacking health-related Euro Quality of Life-Five Dimensions (EQ-5D) data (*n* = 359), pregnant women (*n* = 337), individuals with a history of cancer (*n* = 43), individuals lacking information on household income (*n* = 76), and individuals with daily energy intake below 250 kcal or above 5,000 kcal (*n* = 181). Consequently, the analysis was conducted on a total of 10,224 individuals.

### 2.2 General characteristics

The following variables were subjected to analysis: gender, age, body mass index (BMI), marital status, women’s birth experience, residential area, monthly household income, household composition type, occupation, education level, smoking status, alcohol consumption, and subjective health state of participants. To analyze the monthly household income, the gross household income variable of KNHANES was used. To analyze the annual income, it was divided by 12 in order to obtain the monthly income.

### 2.3 Food security and household income

The investigation of household food security was conducted through the utilization of a question pertaining to the dietary status of the household in question. The participants in the survey were invited to select one of four response options to the question, “Which of the following best describes your household’s dietary life status over the past year?” Those who responded that their family could have a variety of foods as desired were classified as food secure. Those who indicated that their family had sufficient food but could not consume various kinds, or who reported that their family experienced short of food due to financial constraints, were classified as food insecure.

In terms of household income, participants were classified according to quartiles, with those in the low and low-middle categories designated as belonging to the low-income group and those in the middle-high and high categories as belonging to the high-income group. The sensitivity and specificity of a single food insufficiency questionnaire in conjunction with a 18-item food security status questionnaire were reported as 56.8 and 92.3%, respectively (34).

### 2.4 Euro quality of life-five dimensions (EQ-5D)

The EQ-5D was developed by the Euro Quality of Life Group and is an index designed to assess health-related quality of life across five dimensions (35). The instrument comprises five domains: mobility, self-care, usual activity, pain/discomfort, and anxiety/depression. Participants were asked to indicate the extent to which they had problems in each domain on a three-point scale (i.e., not at all, some problems, many problems). The EQ-5D was investigated with the approval of the EuroQol Group^2^, and its validity and reliability were assessed in a population-based survey of the Korean population (35). In this study, participants who responded that their daily life was hindered or that they felt uncomfortable with daily life in the mobility, self-care, usual activity, and pain/discomfort areas, or who responded that they had anxiety or depression in the anxiety/depression area, were classified as experiencing any problems. Participants who responded that they had no discomfort at all in those areas were classified as experiencing no problems.

### 2.5 Assessment of quality of daily meals

#### 2.5.1 Food intake assessment

The food intake of the participants was estimated using the 24-h dietary recall method. In the study, the food items were categorized into 18 food groups, including cereal and cereal products, potatoes and starch products, sugar and sugar products, beans and bean products, nuts and seeds products, vegetables, mushrooms, fruits, meat and meat products, eggs and egg products, fish and shellfish, seaweeds, milk and dairy products, oil and fat, beverages, seasoning, processed foods, and others.

Moreover, the most frequently consumed foods among the participants were identified. In instances where the same food ingredients were used, despite differences in cooking and processing methods, the item was classified as a single food category. The 20 most commonly consumed foods were selected based on the food lists with the highest intake amounts.

#### 2.5.2 Nutrient intake assessment

The daily intake of carbohydrates, protein, and fat for each participant, along with the energy composition, was calculated. The intake of vitamins and minerals (calcium, phosphorus, iron, sodium, potassium, thiamin, riboflavin, niacin, vitamin C, and vitamin A) was evaluated. Calcium and vitamin A are nutrients that are particularly deficient among the Korean population (36, 37). Accordingly, an evaluation of the intake status of these nutrients was conducted. Furthermore, to evaluate the quality of dietary intake, the intake status was investigated in comparison with the 2020 Dietary Reference Intakes for Koreans (38). The proportion of participants who consumed less than the estimated average requirement (EAR) for each nutrient was calculated.

### 2.6 Statistical analysis

The statistical analysis was conducted using the statistical analysis software SAS 9.4 (SAS Institute, Cary, NC, United States) for all the data. In the case of KNHANES, the SURVEY procedure utilizing a stratified, multistage sampling design was implemented, and the significance level was set to *α* = 0.05 for the test. In this study, participants were classified into four study groups based on household food security and income. These were ‘food secure and high income,’ ‘food insecure and high income,’ ‘food insecure and low income,’ and ‘food insecure and low income’ groups. Furthermore, a comparison was conducted between the four groups in terms of general characteristics, eating habits, food and nutrient intake, and the most commonly consumed foods. In addition, the relationship between these variables and health-related quality of life was analyzed. A comparison was made of the general characteristics and eating habits of young adults according to the food security and household income groups, and an estimation was made of the intake of foods and nutrients.

For categorical variables, the ratio (weighted %) considering frequency and weight was calculated by conducting a chi-square test through the SURVEY FREQ procedure. For continuous variables, the weighted mean and standard error were calculated using the SURVEY MEANS procedure, and the significance by groups was tested by conducting an analysis of covariance (ANCOVA) using the SURVEY REG procedure. A *post hoc* analysis was conducted using Tukey’s test, with age and gender as covariates. A logistic regression analysis was conducted to ascertain the odds ratios (ORs) and 95% confidence intervals (CIs) for health-related quality of life in young adults according to food security and household income. The analyses were conducted with adjustments for gender, age, BMI, marital status, residential area, occupation, education level, smoking status, and alcohol consumption. To visually explore the association among the five EQ-5D items (mobility, self-care, usual activities, pain/discomfort, and anxiety/depression), household income, and food security, we performed multiple correspondence analysis, a principal component analysis of nominal data (including weighting variables, but excluding stratification and clustering variables). Correspondence analysis is one of the multidimensional scaling methods, which is an analytical technique that visualizes the correlation between categorical data that can be represented by a row and column split table as an image map in two dimensions for easier understanding (39–41). Therefore, it can be considered a very useful technique for visually illustrating the relationship between categorical data (41, 42). In addition, the explanatory power of two dimensions should be more than 70% to explain the relationship between rows and columns well (39, 40). Statistical analyses were performed using XLSTAT (Addinsoft, France) version 2024.

## 3 Results

### 3.1 General characteristics

A comparison of the demographic characteristics according to household income and food security revealed that all variables except for gender exhibited a statistically significant difference (*p* < 0.05). The “food secure and high income” group exhibited elevated proportions of individuals in the older age range, those with higher monthly household incomes, married persons, university graduates or higher, and administrators/specialists when compared to other groups. Conversely, the “food insecure and low income” group exhibited a higher proportion of high school graduates and current smokers compared to other groups. In addition, the percentage of individuals reporting poor and very poor subjective health status was relatively high within this group (Table 1).

**Table 1:** General characteristics of participants according to the status of household income and food security.

Variables	Food secure and high income (*n* = 3,771)	Food insecure and high income (*n* = 3,041)	Food secure and low income (*n* = 1,380)	Food insecure and low income (*n* = 2,032)	*p*-value^1^
Gender					
Male	1,512 (51.6)	1,251 (53.1)	569 (52.7)	846 (53.3)	0.6134
Female	2,259 (48.4)	1790 (46.9)	811 (47.3)	1,186 (46.7)	
Age (years)	27.2 ± 0.1^a^	27.1 ± 0.1^a^	26.4 ± 0.2^b^	26.0 ± 0.1^b^	<0.0001^2^
BMI (kg/m^2^)	23.0 ± 0.1	22.9 ± 0.1	23.3 ± 0.1	23.3 ± 0.1	0.0387^2^
Marital experience					
Married	1,497 (34.0)	1,103 (29.3)	544 (32.3)	718 (28.5)	0.0009
Single	2,271 (66.0)	1938 (70.7)	832 (67.7)	1,313 (71.5)	
Maternal childbirth experience					
Yes	552 (54.7)	394 (46.2)	263 (65.7)	322 (55.8)	<0.0001
No	419 (45.3)	358 (53.8)	120 (34.3)	205 (44.2)	
Residential area					
Urban	3,367 (90.6)	2,723 (90.9)	1,180 (87.5)	1779 (89.4)	0.0390
Rural	404 (9.4)	318 (9.1)	200 (12.5)	253 (10.6)	
Household structure					
One person	239 (7.8)	133 (5.2)	166 (14.1)	153 (9.3)	<0.0001
Husband and wife	320 (8.7)	178 (5.5)	20 (1.5)	31 (1.5)	
One generation and others	97 (3.1)	109 (4.5)	94 (8.2)	112 (7.0)	
Husband and wife with children	2,357 (60.1)	1874 (58.7)	746 (51.1)	1,032 (47.9)	
Single parent with children	259 (7.5)	349 (12.6)	148 (11.4)	373 (18.5)	
Two generations and others	124 (3.2)	119 (4.0)	64 (4.6)	95 (4.7)	
≥Three generations	375 (9.6)	278 (9.5)	142 (9.1)	236 (11.0)	
Occupation					
Administrators and specialists	911 (24.0)	684 (22.1)	193 (14.6)	260 (12.6)	<0.0001
Clerks	633 (16.6)	514 (16.1)	153 (10.5)	184 (8.6)	
Service workers and marketers	436 (12.2)	395 (13.3)	191 (15.0)	309 (16.8)	
Engineers, technicians, and assemblers	259 (8.1)	233 (9.1)	104 (7.1)	162 (9.0)	
Manual labors	110 (3.3)	121 (5.0)	68 (5.3)	123 (6.3)	
Unemployed (housewife and students)	1,409 (35.9)	1,084 (34.4)	663 (47.6)	982 (46.8)	
Household income (won/month)	559.7 ± 7.5^a^	492.9 ± 6.2^b^	183.3 ± 3.9^c^	174.7 ± 2.6^c^	<0.0001^2^
Educational level					
<High school graduate	42 (1.0)	32 (1.2)	33 (2.4)	100 (4.5)	<0.0001
High school graduate	1,470 (41.6)	1,326 (46.2)	758 (57.2)	1,193 (61.4)	
≥College graduate	2,258 (57.3)	1,682 (52.5)	588 (40.5)	737 (34.1)	
Smoking status					
Current smokers	758 (24.0)	705 (28.4)	334 (27.2)	519 (29.7)	0.0003
Non-smokers or Ex-smokers	3,008 (76.0)	2,333 (71.6)	1,045 (72.8)	1,512 (70.3)	
Alcohol consumption					
≥4 drink/week	108 (3.2)	78 (3.0)	52 (3.8)	73 (4.3)	<0.0001
2–3 drink/week	636 (18.1)	460 (16.2)	229 (17.0)	263 (13.3)	
2–4 drink/month	1,228 (34.3)	1,031 (35.7)	456 (34.8)	653 (34.1)	
1 drink/month	537 (14.0)	427 (14.0)	190 (14.2)	278 (14.0)	
<1 drink/month	798 (20.0)	652 (20.5)	254 (17.8)	409 (18.8)	
Never	455 (10.6)	384 (10.4)	196 (12.4)	352 (15.6)	
Subjective health status					
Very good	236 (6.7)	177 (6.5)	79 (5.8)	111 (6.0)	0.0004
Good	1,479 (38.7)	1,055 (34.7)	478 (34.8)	667 (32.7)	
Average	1,662 (44.4)	1,393 (45.5)	650 (47.0)	946 (46.6)	
Poor	376 (9.7)	395 (12.5)	160 (11.6)	291 (13.9)	
Very poor	18 (0.5)	21 (0.8)	13 (0.8)	17 (0.8)	

Values are *n* (weighted %) or mean ± SE. ^1^*p*-value by *χ*^2^ test, ^2^*p*-value by one-way analysis of variance (ANOVA).

^abc^Different superscripts were significantly different among study groups by Tukey’s test (*α* = 0.05).

### 3.2 Eating habit

Table 2 presents the dietary habits of young adults, classified according to their household income and food security status. The proportion of young adults in the ‘food insecure and low income’ group who skipped breakfast, lunch, and dinner was significantly higher than in the other groups (*p* < 0.05). Young adults in the “food security and high income” group were more likely to eat out than those in other groups, and the percentage of those who used dietary supplements was significantly higher (*p* < 0.05).

**Table 2:** Dietary habit and behavior of subjects according to the status of household income and food security.

Variables	Food secure and high income (*n* = 3,771)	Food insecure and high income (*n* = 3,041)	Food secure and low income (*n* = 1,380)	Food insecure and low income (*n* = 2,032)	*p*-value^1^
Skipping breakfast					
Yes	1,446 (40.6)	1,267 (44.3)	584 (44.0)	860 (44.4)	0.04
No	2,325 (59.4)	1774 (55.7)	796 (56.0)	1,172 (55.6)	
Skipping lunch					
Yes	360 (10.2)	297 (9.9)	152 (11.4)	253 (13.1)	0.01
No	3,411 (89.8)	2,744 (90.1)	1,228 (88.6)	1779 (86.9)	
Skipping dinner					
Yes	216 (5.5)	242 (7.9)	97 (6.9)	169 (8.1)	0.001
No	3,555 (94.5)	2,799 (92.1)	1,283 (93.1)	1862 (91.9)	
Frequency of eating-out					
Everyday	1,480 (43.8)	1,129 (40.7)	423 (35.0)	624 (33.6)	<0.0001
5–6/week	940 (22.8)	837 (25.0)	334 (23.2)	487 (23.2)	
3–4/week	604 (14.9)	483 (14.6)	239 (15.9)	331 (15.9)	
1–2/week	524 (13.0)	430 (14.3)	248 (16.9)	368 (17.3)	
1–3/month	207 (5.2)	149 (5.0)	121 (7.6)	179 (8.1)	
Rarely	15 (0.4)	13 (0.4)	15 (1.3)	42 (2.0)	
Dietary supplement use^2^					
Yes	1,214 (39.9)	844 (37.1)	378 (33.9)	452 (29.6)	<0.0001
No	1716 (60.1)	1,375 (62.9)	681 (66.1)	1,036 (70.4)	

Values are *n* (weighted %). ^1^*p*-value by *χ*^2^ test. ^2^Study subjects with dietary supplement used for longer than 2 weeks during the previous year.

### 3.3 Intake amount of foods and nutrients

Table 3 presents the intake amount by food group in young adults according to household income and food security. The total food intake of the ‘food secure and high income’ group was 1,646.8 g, which was higher than that of the ‘food secure and low income’ and ‘food insecure and low income’ groups but not the ‘food insecure and high income’ group (*p* < 0.05). In the group designated as “food insecure and low income,” the intake of vegetables and beverages was observed to exceed that of the group identified as “food secure and high income.” With regard to fruit, fish and shellfish, and seasoning, the intake of the food security and higher-income group was found to be higher than that of the food security and low-income group, as well as the food insecurity and low-income group, although not the food insecurity and higher-income group. The intake of cereal and cereal products and nuts and seeds products in the food insecurity and low-income group was 295.7 g and 5.3 g, respectively, which were significantly higher than those in the food security and low-income group. The intake of nuts and seeds in the food insecurity and low-income group was significantly higher than that in the food security and low-income group. This was due to the former group consuming acorn jelly (1.98 g) and acorn powder (0.01 g), respectively. Nevertheless, there was no statistically significant difference in nut and seed intake, including almonds, macadamia nuts, and walnuts, between the groups. The data are not presented here.

**Table 3:** Daily food intake of subjects according to the status of household income and food security.

Food group (g)	Food secure and high income (*n* = 3,771)	Food insecure and high income (*n* = 3,041)	Food secure and low income (*n* = 1,380)	Food insecure and low income (*n* = 2,032)	*p*-value^1^
Cereal and cereal products	287.5 ± 3.0^ab^	293.6 ± 3.4^a^	273.9 ± 4.5^b^	295.7 ± 4.3^a^	0.0022
Potatoes and starch products	32.4 ± 1.5^ab^	31.9 ± 1.7^ab^	38.4 ± 2.8^a^	27.0 ± 1.9^b^	0.0103
Sugar and sugar products	11.5 ± 0.4	11.7 ± 0.4	10.0 ± 0.6	10.4 ± 0.5	0.0194
Beans and bean products	35.8 ± 1.6	33.3 ± 1.8	31.5 ± 2.5	30.5 ± 2.7	0.3167
Nuts and seeds products	4.3 ± 0.3^a^	4.6 ± 0.5^a^	2.7 ± 0.3^b^	5.3 ± 1.1^a^	0.0014
Vegetables	278.9 ± 3.9^a^	270.7 ± 4.2^ab^	263.5 ± 6.5^ab^	251.4 ± 5.0^b^	0.0091
Mushrooms	6.0 ± 0.3	6.7 ± 0.7	5.2 ± 0.4	5.3 ± 0.4	0.2231
Fruits	160.9 ± 5.3^a^	150.5 ± 5.4^ab^	126.2 ± 6.8^bc^	115.0 ± 5.3^c^	<0.0001
Meat and meat products	145.0 ± 3.4	140.9 ± 3.8	138.4 ± 5.1	133.0 ± 4.7	0.0958
Eggs and egg products	31.4 ± 0.9	33.0 ± 1.0	31.4 ± 1.5	31.7 ± 1.3	0.6840
Fish and shellfish	74.9 ± 2.1^a^	68.9 ± 2.7^ab^	62.1 ± 2.8^b^	60.2 ± 3.3^b^	0.0015
Seaweeds	12.6 ± 1.1	12.4 ± 1.5	8.9 ± 1.1	12.0 ± 2.0	0.1395
Milk and dairy products	110.0 ± 3.4	115.6 ± 4.3	104.1 ± 5.6	110.9 ± 5.3	0.2891
Oil and fat	10.2 ± 0.2	10.1 ± 0.2	9.7 ± 0.4	9.7 ± 0.3	0.3973
Beverages	389.2 ± 10.5^a^	375.6 ± 11.4^ab^	386.2 ± 17.6^ab^	341.8 ± 13.1^b^	0.0224
Seasoning	42.7 ± 0.9^a^	39.7 ± 1.0^ab^	37.8 ± 1.2^b^	37.7 ± 1.1^b^	0.0010
Processed foods	13.2 ± 1.2	9.7 ± 1.0	11.4 ± 1.6	10.0 ± 1.4	0.0842
Other	0.4 ± 0.1	0.3 ± 0.1	0.5 ± 0.2	0.2 ± 0.1	0.3525
Total food intake	1646.8 ± 15.7^a^	1609.2 ± 17.2^ab^	1541.9 ± 26.5^bc^	1487.8 ± 20.8^c^	<0.0001

Values are mean ± SE. ^1^Significant difference among study groups was analyzed by ANCOVA including age and gender as covariates. ^abc^Different superscripts were significantly different among study groups by Tukey’s test (*α* = 0.05).

The food security and higher-income group exhibited higher intake of energy, fat, calcium, phosphorus, iron, potassium, riboflavin, and vitamin C compared to the food insecurity and low-income group (Table 4). In addition, the food security and higher-income group exhibited a lower intake of energy from carbohydrates, while their intake of energy from fat was higher, in comparison with the food insecurity and low-income group.

**Table 4:** Daily energy and nutrient intake of subjects according to the status of household income and food security.

Variables	Food secure and high income (*n* = 3,771)	Food insecure and high income (*n* = 3,041)	Food secure and low income (*n* = 1,380)	Food insecure and low income (*n* = 2,032)	*p*-value^1^
Energy (kcal)	2136.9 ± 16.6^a^	2143.1 ± 19.8^a^	2049.6 ± 28.4^bc^	2066.1 ± 23.1^b^	0.0030
Carbohydrate (g)	302.4 ± 2.4^ab^	306.3 ± 2.7^a^	292.0 ± 3.8^b^	297.7 ± 3.3^ab^	0.0322
Protein (g)	79.3 ± 0.8	79.7 ± 0.9	76.3 ± 1.3	75.9 ± 1.2	0.0222
Fat (g)	57.1 ± 0.7^a^	55.9 ± 0.8^ab^	53.5 ± 1.2^b^	53.4 ± 0.9^b^	0.0005
Calcium (mg)	506.7 ± 5.5^a^	513.1 ± 6.8^a^	479.2 ± 9.9^ab^	471.5 ± 7.7^b^	0.0005
Phosphorus (mg)	1158.8 ± 9.4^a^	1171.7 ± 11.7^a^	1113.5 ± 16.9^ab^	1098.4 ± 13.7^b^	0.0005
Iron (mg)	14.3 ± 0.2^a^	14.4 ± 0.2^a^	13.2 ± 0.3^b^	12.9 ± 0.3^b^	<0.0001
Sodium (mg)	4396.9 ± 53.7^a^	4413.7 ± 62.1^a^	4090.0 ± 79.0^b^	4230.5 ± 68.4^ab^	0.0188
Potassium (mg)	2893.2 ± 25.5^a^	2884.2 ± 30.1^ab^	2733.6 ± 42.1^bc^	2665.1 ± 35.9^c^	<0.0001
Thiamin (mg)	1.6 ± 0.0	1.6 ± 0.0	1.5 ± 0.0	1.6 ± 0.0	0.0517
Riboflavin (mg)	1.5 ± 0.0^a^	1.5 ± 0.0^a^	1.4 ± 0.0^b^	1.4 ± 0.0^b^	0.0007
Niacin (mg)	16.9 ± 0.2	17.3 ± 0.2	16.4 ± 0.3	16.2 ± 0.3	0.0579
Vitamin C (mg)	86.8 ± 1.8^a^	86.3 ± 2.1^a^	80.8 ± 2.5^ab^	76.4 ± 2.2^b^	0.0067
Vitamin A (μgRE)	737.7 ± 16.9	767.2 ± 23.5	716.7 ± 37.6	676.4 ± 20.5	0.0826
Energy from carbohydrate (%)	61.7 ± 0.2^b^	62.4 ± 0.2^ab^	62.5 ± 0.4^ab^	62.9 ± 0.3^a^	0.0003
Energy from protein (%)	14.9 ± 0.1	14.8 ± 0.1	14.9 ± 0.1	14.6 ± 0.1	0.1450
Energy from fat (%)	23.4 ± 0.2^a^	22.8 ± 0.2^ab^	22.6 ± 0.3^b^	22.5 ± 0.2^b^	0.0006

Values are mean ± SE. ^1^Significant difference among study groups was analyzed by ANCOVA including age and sex as covariates. ^abc^Different superscripts were significantly different among study groups by Tukey’s test (*α* = 0.05).

### 3.4 Proportion of participants who consumed less than the estimated average requirement (EAR)

The percentages of young adults who consumed less than the EAR for nutrients were compared, and the results are presented in Table 5. The percentage of young adults in the food insecure and low income groups who consumed less than the EAR for protein, vitamin A, riboflavin, niacin, vitamin C, calcium, and iron was higher than that of all other groups (*p* < 0.05). The percentage of young adults in the food insecure and low income groups who consumed less than the EAR for vitamin A was higher than that of other groups. With regard to phosphorus, the proportion of subjects who consumed less than the EAR was greater in the low-income groups than in those of higher income. With regard to vitamin A, vitamin C, and calcium, over 50% of young adults in the food insecurity and low-income group consumed quantities below the EAR. These figures were 53.6, 65.9, and 74.4%, respectively.

**Table 5:** Percentage of subjects consuming nutrients less than estimated average requirement (EAR) from daily diet according to the status of household income and food security.

Nutrients	Food secure and high income (*n* = 3,771)	Food insecure and high income (*n* = 3,041)	Food secure and low income (*n* = 1,380)	Food insecure and low income (*n* = 2,032)	*p*-value^1^
		%			
Carbohydrate	2.1	2.3	2.8	2.6	0.4477
Protein	18.2	20.2	23.2	25.2	<0.0001
Vitamin A	47.6	46.8	51.8	53.6	<0.0001
Thiamin	24.4	26.3	29.7	27.9	0.0035
Riboflavin	36.3	38.1	42.4	43.8	<0.0001
Niacin	30.3	30.6	34.7	36.2	<0.0001
Vitamin C	59.6	60.9	62.1	65.9	0.0009
Calcium	71.3	70.2	73.7	74.4	0.0172
Phosphorus	10.1	10.7	13.1	13.1	0.0034
Iron	33.9	34.0	38.7	39.8	<0.0001

^1Significant differences among the study groups were analyzed by χ2 test.^

### 3.5 Most commonly consumed food

Table 6 shows the results of the calculation of commercial foods based on the intake amounts of the foods in question. The food insecurity and low-income group consumed the greatest quantity of rice, milk, and beer, while the other groups consumed rice, beer, and milk in descending order of quantity. In the food insecurity and low-income group, ramen was ranked 11th, which was relatively higher than other groups, and beef and fruit were not included within the 15th place rank. In contrast, the apple was the 11th most consumed food in both the food security and higher-income group and the food insecurity and higher-income group, respectively, while beef was in 13th and 14th place in the same two aforementioned groups. It is noteworthy that Sprite was ranked 12th in the food insecurity and low-income group, 14th in the food security and low-income group, and 18th in the food insecurity and higher-income group but not even on the food security and higher-income group.

**Table 6:** Most consumed food items of subjects according to the status of household income and food security.

Rank	Food secure and high income (*n* = 3,771)				Food insecure and high income (*n* = 3,041)				Food secure and low income (*n* = 1,380)				Food insecure and low income (*n* = 2,032)			
	Food	Intake (g)	Intake (%)	CP^1^ (%)	Food	Intake (g)	Intake (%)	CP (%)	Food	Intake (g)	Intake (%)	CP (%)	Food	Intake (g)	Intake (%)	CP (%)
1	Rice	137.23	8.46	8.46	Rice	145.73	9.20	9.20	Rice	143.34	9.39	9.39	Rice	149.10	10.08	10.08
2	Beer	95.85	5.91	14.36	Beer	85.87	5.42	14.62	Beer	100.73	6.60	15.99	Milk	78.97	5.34	15.42
3	Milk	76.66	4.72	19.09	Milk	81.07	5.12	19.73	Milk	67.60	4.43	20.42	Beer	76.93	5.20	20.63
4	Kimchi	61.52	3.79	22.88	Kimchi	63.99	4.04	23.77	Kimchi	62.06	4.07	24.49	Kimchi	66.81	4.52	25.15
5	Pork	55.06	3.39	26.27	Pork	54.08	3.41	27.18	Pork	58.95	3.86	28.35	Cola	54.80	3.71	28.85
6	Cola	48.60	2.99	29.27	Cola	48.31	3.05	30.23	Cola	56.43	3.70	32.05	Pork	53.29	3.60	32.46
7	Soju	38.38	2.37	31.63	Soju	40.80	2.58	32.81	Chicken	43.16	2.83	34.88	Chicken	40.54	2.74	35.20
8	Chicken	37.62	2.32	33.95	Chicken	39.66	2.50	35.31	Soju	37.39	2.45	37.33	Soju	40.31	2.73	37.92
9	Egg	31.77	1.96	35.91	Egg	33.15	2.09	37.40	Egg	31.69	2.08	39.41	Egg	32.23	2.18	40.10
10	Onion	31.53	1.94	37.85	Onion	30.69	1.94	39.34	Onion	31.50	2.06	41.47	Onion	28.61	1.94	42.04
11	Apple	28.02	1.73	39.58	Apple	26.90	1.70	41.04	Potato	29.66	1.94	43.41	Ramen	25.80	1.74	43.78
12	Green tea^1^	25.95	1.60	41.17	Fruit drink	23.51	1.48	42.52	Green tea^1^	25.35	1.66	45.07	Sprite	25.18	1.70	45.49
13	Beef	25.05	1.54	42.72	Potato	23.39	1.48	44.00	Apple	22.76	1.49	46.57	Bread	21.13	1.43	46.91
14	Fruit drink	24.65	1.52	44.24	Beef	23.19	1.46	45.46	Sprite	21.90	1.44	48.00	Potato	19.26	1.30	48.22
15	Bread	24.18	1.49	45.73	Ramen	20.77	1.31	46.77	Fruit drink	20.54	1.35	49.35	Chili	19.25	1.30	49.52
16	Potato	23.52	1.45	47.18	Bread	20.76	1.31	48.08	Bread	20.06	1.31	50.66	Fruit drink	19.16	1.30	50.82
17	Mandarin	21.04	1.30	48.47	Chili	20.60	1.30	49.38	Mandarin	19.96	1.31	51.97	Mandarin	18.42	1.25	52.06
18	Beef bone soup	20.70	1.28	49.75	Sprite	19.78	1.25	50.63	Cucumber	19.69	1.29	53.26	Green tea^2^	18.36	1.24	53.30
19	Chili	20.40	1.26	51.00	Mandarin	19.37	1.22	51.85	Beef	18.77	1.23	54.49	Apple	18.28	1.24	54.54
20	Tofu	19.89	1.23	52.23	Green tea^1^	19.10	1.21	53.06	Chili	18.51	1.21	55.70	Beef	17.44	1.18	55.72

^1CP, Cumulative percent. 2Green tea infusion.^

### 3.6 Relationship among household income, food security, and health-related quality of life

Table 7 presents the OR and 95% CI for the index of health-related quality of life among young adults stratified by household income and food security status. The prevalence of any mobility problems was 1.55 times higher in the low-income group than in the food secure and high income group [OR (95% CI) = 1.55 (1.05–2.29)]. Similarly, the prevalence of anxiety/depression was 1.33 times higher in the low-income group than in the food secure and high income group [OR (95% CI) = 1.33 (1.07–1.64)]. Nevertheless, no significant correlation was observed in the domains of self-care, usual activity, and pain/discomfort.

**Table 7:** ORs and 95% CIs of EQ-5D^1^ according to the status of household income and food security.

Variables	Food secure and high income (*n* = 3,771)	Food insecure and high income (*n* = 3,041)	Food secure and low income (*n* = 1,380)	Food insecure and low income (*n* = 2,032)	*p*-value
Mobility					
Any problem, *n* (%)^2^	80 (2.0)	76 (2.4)	33 (2.3)	74 (3.7)	0.0020^3^
Age- and sex-adjusted	Ref	1.24 (0.86–1.79)	1.19 (0.75–1.91)	1.98 (1.39–2.83)	
Multiple adjusted^3^	Ref	1.22 (0.84–1.76)	1.04 (0.64–1.68)	1.55 (1.05–2.29)	
Self-care					
Any problem, *n* (%)	16 (0.5)	11 (0.3)	3 (0.2)	15 (0.7)	0.0677
Age- and sex-adjusted	Ref	0.64 (0.28–1.46)	0.32 (0.08–1.34)	1.49 (0.68–3.28)	
Multiple adjusted	Ref	0.68 (0.29–1.58)	0.21 (0.04–1.18)	1.11 (0.50–2.49)	
Usual activity					
Any problem, *n* (%)	64 (1.9)	62 (1.9)	28 (1.8)	52 (2.8)	0.1512
Age- and sex-adjusted	Ref	1.01 (0.68–1.48)	1.00 (0.58–1.66)	1.51 (1.00–2.30)	
Multiple adjusted	Ref	1.00 (0.67–1.48)	0.85 (0.50–1.45)	1.25 (0.81–1.94)	
Pain/discomfort					
Any problem, *n* (%)	496 (13.2)	437 (13.7)	191 (13.6)	301 (14.3)	0.7708
Age- and sex-adjusted	Ref	1.05 (0.90–1.22)	1.05 (0.86–1.28)	1.12 (0.94–1.34)	
Multiple adjusted	Ref	1.04 (0.90–1.22)	0.97 (0.79–1.19)	1.01 (0.84–1.22)	
Anxiety/depression					
Any problem, *n* (%)	283 (7.5)	254 (7.8)	114 (7.8)	236 (10.6)	0.0010
Age- and sex-adjusted	Ref	1.06 (0.87–1.29)	1.05 (0.81–1.35)	1.46 (1.19–1.79)	
Multiple adjusted	Ref	1.05 (0.86–1.28)	0.99 (0.76–1.27)	1.33 (1.07–1.64)	

^1^OR, odds ratio; CI, confidence interval; EQ-5D, Euro Quality of Life-Five Dimensions. ^2^Proportions are given as *n* (%) reporting any problems. ^3^*p*-value by *χ*^2^ test. ^3^The multiple models were adjusted for age, sex, BMI, marital status, residential area, occupation, education level, smoking status, and alcohol consumption.

**Figure 1:**
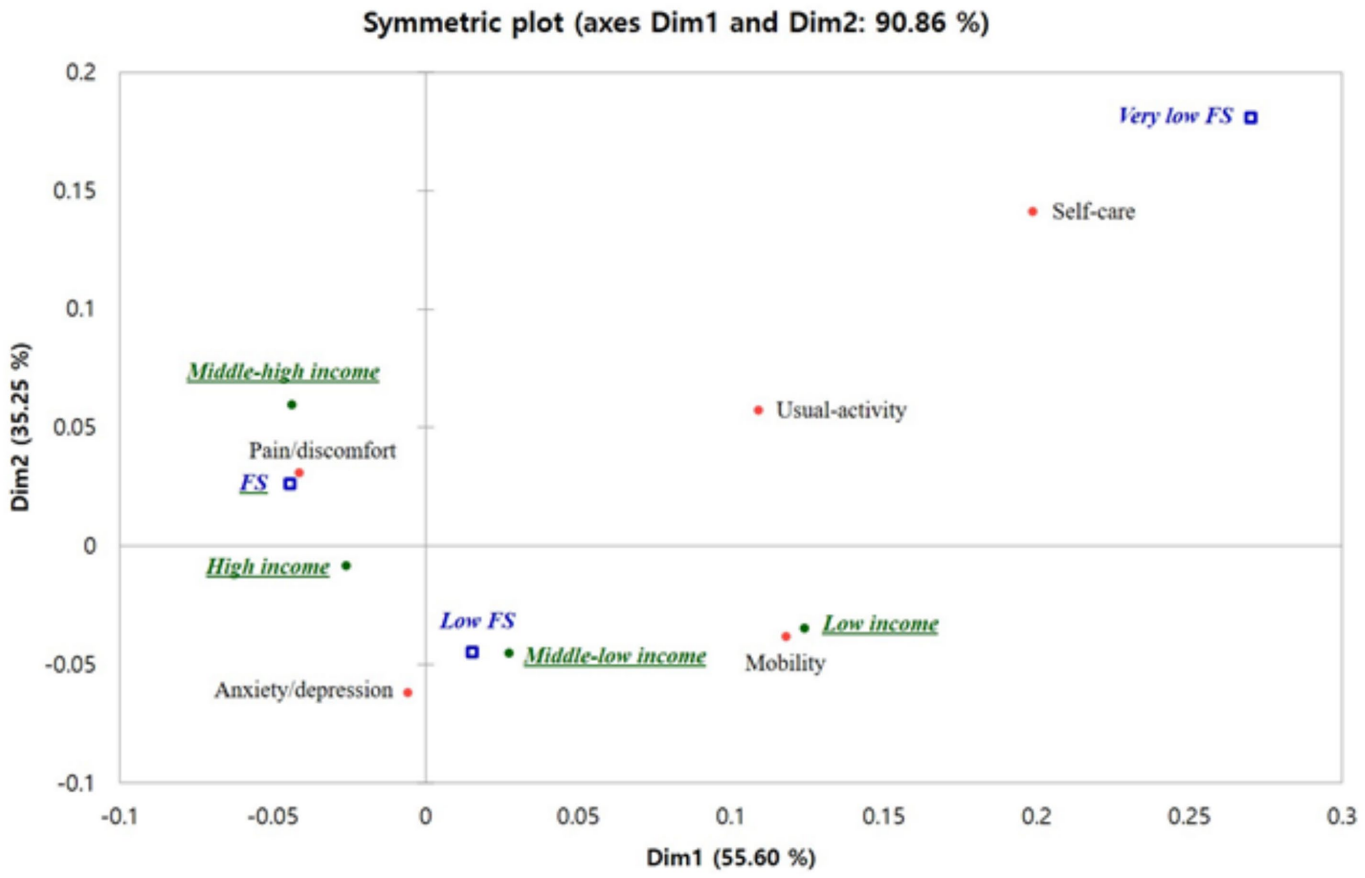
Result of multiple correspondence analysis for relationship among food security, household income, and the EQ-5D components. FS, food security. Food secure: the family was able to consume sufficient quantity of food and a diverse range of food types. Low food secure: the family was able to consume sufficient quantity of food yet lacked the diversity of food types. Very low food secure: the family experienced financial difficulties, leading to a lack of sufficient food. EQ5D components are having some or a lot of problems with mobility, self-care, usual activity, pain/discomfort, and anxiety/depression.

The results of multiple correspondence analysis, a principal component analysis of nominal data, to analyze the relationship among food security, household income, and the components of the EQ-5D are shown in Figure 1. Dim1 (55.6%) and Dim2 (35.26%) had an explanatory power of 90.86%, showing good explanatory power of more than 70%. Very low FS was located in the first quadrant and was highly correlated with self-care and usual activity. Next, FS was located in quadrant 2 and was closely associated with pain/discomfort and middle-high income. In the third quadrant, high income and anxiety/depression were closely related, and finally, low_FS was in the fourth quadrant, closely related to low and middle-low income and mobility.

## 4 Discussion

This cross-sectional study aimed to evaluate the dietary quality of young adults aged 19–34 years and ascertain whether there was a relationship between dietary habits and the decline of health-related quality of life. The study made use of KNHANES data from 2008 to 2018, which included a vulnerable group that experienced particular difficulties in food-related life. It was confirmed that the food insecure and low income group had lower intakes of food and nutrients, including fruits, vegetables, fish and shellfish, protein, some vitamins, and minerals, in comparison with the food secure and high income group. In the category of commonly consumed foods, ramen and Sprite were found to be relatively high in the food insecurity and low-income group, while apple and beef were identified as being high in the food security and higher-income group. Furthermore, we observed that individuals in the food insecure and low income group exhibited a 55 and 33% increased likelihood of experiencing mobility issues and anxiety/depression, respectively.

The present study revealed that the food insecurity and low-income group exhibited lower levels of total food intake, energy intake, and diet quality compared to the food security and higher-income group. The intake of cereal and cereal products was high, whereas the intake of vegetables, fruits, fish, and shellfish was significantly low in the food insecurity and low-income group. In addition, prior research has documented a correlation between food insecurity and lower fruit and vegetable intake (11, 43). A negative association was observed between household food insecurity and intakes of protein, all vitamins, and minerals in the Canadian Community Health Survey (4). Conversely, a higher energy density and a greater proportion of energy derived from carbohydrates were found to be positively associated with household food insecurity. The purchase of nutritionally beneficial, yet expensive food items may be constrained by financial considerations (44). It appears that food insecurity is associated with limited accessibility to fresh foods, such as vegetables and fruits (45–47). In this study, there were notable differences in income level, residential area, and household composition type according to food security status. In particular, the groups identified as experiencing food insecurity displayed a high prevalence of single parents with unmarried children. It is hypothesized that these socio-economic characteristics of the household are associated with differences in lifestyle, including dietary intake.

These findings were in accordance with those of previous studies. The preceding studies (6, 48–50) demonstrated that there were more younger people, women, and single parents with children in the food insecure household group than in the food secure household group. In addition, the former group lacked home ownership and exhibited a low income. Moreover, lower income, socio-economic status, and education level are associated with higher dietary energy density and lower diet quality (51, 52). The observed discrepancy in dietary quality was attributed to an elevated consumption of added sugars, sodium, and saturated fats, coupled with a diminished intake of fruits and vegetables. In the present study, the food insecure and low income group demonstrated a higher intake of ramen and Sprite compared to other groups. Previous studies have indicated that food insecurity is associated with a reduction in dietary quality (53–55). Nutrient-dense foods, including fruits and vegetables, are frequently more costly and less accessible to those in low-income groups than processed foods. Processed foods are typically inexpensive and readily available.

In this study, the percentage of participants who consumed less than the EAR of KDRI (38) was calculated to assess the diet quality of the subjects. Consequently, the food insecure and low income cohort exhibited a higher proportion of participants who consumed less than the EAR for nutrients, with the exception of carbohydrates. With regard to vitamin A, vitamin C, and calcium, the proportion of participants who consumed less than the EAR was in excess of 50%. In particular, calcium was the micronutrient for which participants exhibited the greatest insufficiency, with 74.4% of young adults in the food insecurity and low-income group taking less than the EAR (*p* < 0.0172). In contrast, no significant difference in calcium intake was observed between US adults according to their food security status (54). The calcium intake of the participants in this study was found to be 471.5–406.7 mg, while the calcium intake of US women aged 20–70 years between 1999 and 2000 was 756 mg (55). This indicates that the calcium intake of the participants in this study was overall lower than that of US women, with the participants in the food insecurity and low-income group exhibiting the lowest calcium intake. A systematic review of the literature reveals that the majority of countries in Asia have daily dietary calcium intakes below 500 mg (56). Furthermore, the present study revealed that the calcium intake among Korean adults exhibited variation according to income level, whereas in Brazil, this trend was not observed. In our study, although milk was the second and third most consumed food item, the consumption of milk and dairy products, which are major sources of calcium, appeared to be lower than that observed in Western countries.

As in previous studies (8, 24), this study corroborated the hypothesis that there is a correlation between food insecurity and diminished health-related quality of life. In particular, the prevalence of mobility problems and anxiety/depression was significantly increased in the dietary life of the vulnerable group, namely, the food insecure and low income group, as evidenced by this study. A substantial body of research has documented the association between food insecurity and mental health outcomes (3, 57, 58). A meta-analysis of the relationship between food insecurity and mental health was conducted using data from 19 studies conducted in 10 different countries. As a result, food insecurity was found to increase the risk of depression in adults by 1.44 times <OR (95% CI) = 1.44 (1.30–1.58) and stress by 1.34 times <OR (95% CI) = 1.34 (1.24–1.44) (17).

It should be noted that this study has certain limitations. As the KNHANES is a cross-sectional study, it was not possible to confirm the causal relationship between EQ-5Ds and income and food security. Moreover, the use of a single 24-h dietary recall may not be sufficient to estimate usual dietary intake. In addition, it is not feasible to assess the absence of nutrient intake with complete precision using the EAR method. Accordingly, the findings of this study must be interpreted as a relative assessment of groups stratified by food security status and income level. In addition, food security was assessed via a single question regarding household food insufficiency, which may not be an optimal method for measuring food security status. The use of a single item for measuring food security status may result in an underestimation of the prevalence of food insecurity, due to the low sensitivity of the measure (59). A validation study conducted in Korea assessed the sensitivity and specificity of the single-item question used in the KNHANES in conjunction with the food security measures developed based on the US Household Food Security Survey Module (34). The sensitivity and specificity of the food insufficiency question were found to be 56.8 and 92.3%, respectively. This finding is consistent with those of previous studies (59–61). However, Urke et al. (62) have indicated that a single question for measuring food security could prove a useful tool in a large-scale investigation in terms of rapid assessment. However, this study has the advantage of being a large-scale population-based investigation, the first to analyze the relationship between household income and food security and EQ-5D in Korean young adults.

The following conclusions and recommendations emerge from the findings of this study. Income level and food insecurity status in Korean young adults were found to be associated with dietary intake status and health-related quality of life, particularly in relation to mobility and anxiety/depression. It is therefore imperative that measures be taken to support nutrients for this vulnerable group in dietary life and improve their accessibility to healthy and fresh food. Furthermore, the findings of this study can serve as a foundation for the formulation of government policies aimed at reducing disparities and inequalities in dietary habits and health outcomes among young adults.

## Data Availability

The original contributions presented in the study are included in the article/supplementary material, further inquiries can be directed to the corresponding author.

## Footnotes

^1^ http://knhanes.kdca.go.kr

^2^ www.euroqol.org
